# β-Carboline Alkaloids and Essential Tremor: Exploring the Environmental Determinants of One of the Most Prevalent Neurological Diseases

**DOI:** 10.1100/tsw.2010.159

**Published:** 2010-09-01

**Authors:** Elan D. Louis, Wei Zheng

**Affiliations:** ^1^ GH Sergievsky Center, College of Physicians and Surgeons, Columbia University, New York, NY, USA; ^2^ Department of Neurology, College of Physicians and Surgeons, Columbia University, New York, NY, USA; ^3^ Taub Institute for Research on Alzheimer's Disease and the Aging Brain, College of Physicians and Surgeons, Columbia University, New York, NY, USA; ^4^ Department of Epidemiology, Mailman School of Public Health, Columbia University, New York, NY, USA; ^5^ school of Health Sciences, Purdue University, West Lafayette, IN, USA

**Keywords:** essential tremor, environmental epidemiology, etiology, toxin, harmane

## Abstract

Essential tremor (ET) is among the most prevalent neurological diseases, yet its etiology is not well understood. Susceptibility genotypes undoubtedly underlie many ET cases, although no genes have been identified thus far. Environmental factors are also likely to contribute to the etiology of ET. Harmane (1-methyl-9H-pyrido[3,4-β]indole) is a potent, tremor-producing β-carboline alkaloid, and emerging literature has provided initial links between this neurotoxin and ET. In this report, we review this literature. Two studies, both in New York, have demonstrated higher blood harmane levels in ET cases than controls and, in one study, especially high levels in familial ET cases. Replication studies of populations outside of New York and studies of brain harmane levels in ET have yet to be undertaken. A small number of studies have explored several of the biological correlates of exposure to harmane in ET patients. Studies of the mechanisms of this putative elevation of harmane in ET have explored the role of increased dietary consumption, finding weak evidence of increased exogenous intake in male ET cases, and other studies have found initial evidence that the elevated harmane in ET might be due to a hereditarily reduced capacity to metabolize harmane to harmine (7-methoxy-1-methyl-9H-pyrido[3,4-β]-indole). Studies of harmane and its possible association with ET have been intriguing. Additional studies are needed to establish more definitively whether these toxic exposures are associated with ET and are of etiological importance.

## INTRODUCTION

Essential tremor (ET) is a chronic neurological disease that is usually progressive. Its hallmark clinical feature is a 4- to 12-Hz kinetic tremor (i.e., tremor that occurs during volitional movements) of the arms[1], which may manifest during a broad range of daily tasks (e.g., eating, writing, and shaving) (Fig. 1). Across affected individuals, this tremor may range in severity from mild and functionally inconsequential to severe and disabling[2,3,4,5]. In addition to the classical kinetic tremor of the arms, patients may exhibit a variety of tremors that differ by location (e.g., head, voice, and jaw) and condition (postural, intentional, and rest)[1,6,7,8]. Aside from tremor, other motor manifestations have been documented; perhaps most notable of these is a gait that has been described as mildly ataxic[9,10]. In addition, there is growing recognition of the presence of mild cognitive changes (especially executive dysfunction)[11,12,13,14,15,16]. In epidemiological studies, ET has been shown to be associated with increased risks of Alzheimer's disease[17,18] and Parkinson's disease[19]. Although traditionally viewed as a disease of morbidity rather than mortality, in the single, prospective, population-based study that enrolled a control group, there was a 45% increased risk of mortality[20], despite the absence of such a finding in one earlier retrospective study using historical controls[21].

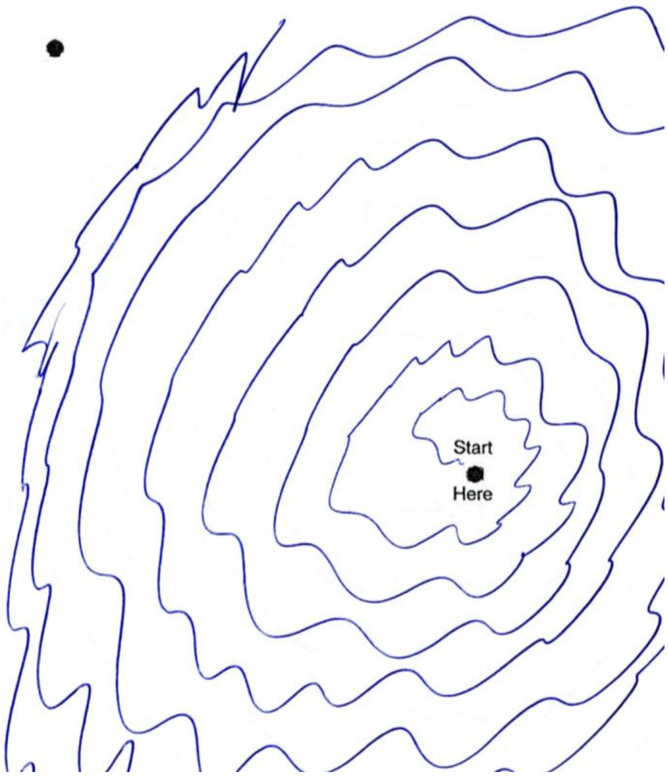

**FIGURE 1.** The effects of tremor are apparent in an ET patient's attempt to draw an Archimedes spiral.

ET is one of the most common neurological diseases; in a meta-analysis of the 28 population-based prevalence studies, the pooled prevalence (all ages) = 0.9%[22]. A commonly cited value is that the population prevalence is 4.0% after age 39 years[23]. The prevalence increases markedly with age, especially with advanced age (i.e., 4.6% among persons aged 65 years and older, and as high as 21.7% in persons aged 95 and older)[22].

The underlying pathogenesis of ET is poorly understood, although recent postmortem studies indicate the presence of degenerative changes in the cerebellum in the majority of cases, including Purkinje cell loss in excess of that seen in similarly aged controls[24,25].

## ETIOLOGY OF ET

The common presence, in clinic samples, of ET families with multiple affected individuals over several generations has led to the view that genetic susceptibility is of importance in disease etiology[26]. Although no genes have yet been identified, linkage to regions on chromosome 2p[27], 3q[28], and 6p[29] indicate that genetic factors are indeed of importance in this disease. A commonly cited value is that 50% of ET cases occur on a familial basis[30,31].

Although widely considered to have a large genetic component, it is likely that environmental factors also contribute to the etiology of ET[32]. The high preponderance of ET cases without affected relatives (>50% of ET cases in some series)[31,33,34,35,36,37,38] and the results from concordance studies (60% concordance in monozygotic twins in one study and 63% in another)[39,40] are among the observations that have been used to support the notion that nongenetic factors are important in this disease[32,41]. The existence of intrafamilial differences in age of onset and severity of tremor[42,43] also suggests that environmental (or perhaps other genetic) factors may be serving as modifiers of underlying susceptibility genotypes.

Environmental factors are also thought to play a substantial role in other neurological disorders (Parkinson’s disease, Alzheimer’s disease, amyotrophic lateral sclerosis)[44,45,46,47,48,49,50,51,52, 53,54,55], so that it is not inconceivable that they could play a role in ET. Using the commonly cited value of 50% for the percentage of cases that occur on a familial basis[30,31] and given its population prevalence of 4.0% after age 39 years[23], this then suggests that approximately 2.0% of the population aged ≥40 years has a nonfamilial form of ET[23,32]. Despite the apparent extent of the problem, the environmental correlates for this tremor are just beginning to be explored. One possible link has been established with the exposure to β-carboline alkaloids. The purpose of this article is to critically review the published literature on the links between β-carboline alkaloids and ET.

## β-CARBOLINES AS POSSIBLE ENVIRONMENTAL TOXINS IN ET

### Rationale for Their Study in ET

The β-carboline alkaloids are a group of naturally occurring, tremor-producing chemicals that include harmane, harmine, harmaline, and several others[32,56,57,58]. Structurally, the β-carboline alkaloids are heterocyclic amines, comprised of a combination of five- and six-ringed (i.e., cyclic) carbon structures, containing an amine group[59,60] (Fig. 2). There is considerable structural similarity with 1-methyl-4-phenyl-1,2,3,6-tetrahydropyridine (MPTP), which is used to produce one of the main toxin-induced animal models for Parkinson’s disease[61,62,63]. Like MPTP, the β-carboline alkaloids are highly neurotoxic, and the administration of β-carboline alkaloids to a wide variety of laboratory animals produces an intense and generalized action tremor that resembles ET[32,64]. Indeed, β-carboline alkaloid administration is currently viewed as the main animal model for ET and new pharmacotherapies are tested using exposed animals[65,66,67,68,69]. The tremor that is produced by β-carboline alkaloids shares several features with ET, including its primary clinical features and drug-response characteristics[69,70,71,72,73,74], and underlying brain changes (destructive changes in the cerebellum, including Purkinje cell loss, which has now been documented in ET and in the β-carboline alkaloid model of tremor)[25,64,69,70,75,76,77,78,79]. Human volunteers exposed to intravenously administered harmine exhibit neurological signs, including an acute, coarse tremor[80,81].

Harmane (1-methyl-9H-pyrido[3,4-β]indole) is a potent, tremor-producing β-carboline alkaloid[56], capable of inducing tremor in laboratory animals within 3.1 min of subcutaneous injection[57]. Due to its high lipid solubility, harmane accumulates in brain tissue, with brain concentrations in laboratory animals 6.5 times those injected into peripheral tissues[57]. Furthermore, harmane is one of the most abundant of all dietary heterocyclic amines and human exposure to harmane through diet is greater than that of other heterocyclic amines[82,83], making it a β-carboline alkaloid of particularly high interest in epidemiological studies of humans[32].

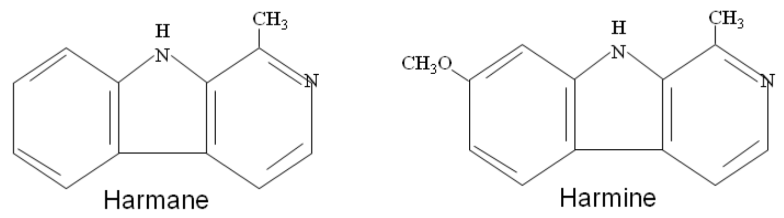

**FIGURE 2.** Chemical structures of harmane (1-methyl-9H-pyrido[3,4-β]indole) and harmine (7-methoxy-1-methyl-9H-pyrido[3,4-β]-indole).

β-Carboline alkaloids are produced by the body endogenously[84,85], although exogenous sources are likely to be more important; one study estimated that dietary sources were 50 times greater than these endogenous sources[86]. β-Carboline alkaloids may be found in particularly high ng/g concentrations in animal protein (i.e., meat) and cooking results in additional increases in concentrations[83,87,88,89,90]. β-Carboline alkaloids are also present in varying concentrations in a variety of plant-derived foods/substances, including coffee, ethanol, and tobacco[32,91]. The demonstrated tremor-inducing property of the β-carboline alkaloids, along with their presence in the food supply, provides the rationale for an in-depth scrutiny of their potential role in the etiology of ET, the most common tremor disorder.

## Are Levels Elevated in ET?

Hypothesizing that β-carboline alkaloids might be an environmental exposure of etiological importance in ET, Louis and colleagues began to study their blood concentrations in ET cases in 2000[60,92]. Concentrating their efforts on harmane, which is one of the most abundant of all dietary heterocyclic amines[82,83], and harmine (7-methoxy-1-methyl-9H-pyrido[3,4-β]-indole), a probable metabolic breakdown product of harmane[60], they demonstrated that blood harmane concentration was elevated in 100 ET cases at the Neurological Institute of New York compared to 100 matched control subjects (median blood harmane [cases] = 5.21 vs. 2.28 $g^{-10}$/mL [controls], $p = 0.005$)[93]. Blood harmine concentrations were also marginally, but not significantly, elevated in ET cases (median blood harmine [cases] = 1.60 vs. 0.65 $g^{-10}$/mL [controls], $p = 0.19$). A larger replicate sample of 150 ET cases and 135 matched controls was assembled over the ensuing 5 years (2002–2007)[94]; while overall concentrations were lower in that sample, the case-control difference persisted (median blood harmane [cases] = 2.61 vs. 1.82 $g^{-10}$/mL [controls], $p = 0.016$)[94]. Among ET cases, there was no correlation between blood harmane concentration and tremor severity (total tremor score, Pearson's $r = -0.01$, $p = 0.87$), yet in a small subgroup of 24 ET cases with very severe tremor (total tremor score ≥25), blood harmane concentrations were very high. In linear regression analysis in which log blood harmane concentration was the outcome variable, the ordinal independent variable was coded as follows: ET with high total tremor score (2), remainder of ET cases (1), and controls (0), beta = 0.16, $p = 0.03$, indicating a significant trend (i.e., as one progressed from controls to ET cases and finally to ET cases with severe tremor, blood harmane concentration progressively increased)[94].

These two studies provide preliminary evidence for elevated blood harmane concentrations in ET. There is, however, a need to replicate these data at other centers with additional ET case samples.

## Are Levels Elevated in the ET Brain?

Levels of harmane have been shown to be elevated in the blood in two studies to date. This result is intriguing; however, the brain is the presumptive target organ for harmane-induced effects in patients with

ET. Are brain levels of harmane elevated in ET? Data are indirect and very limited and will be reviewed below.

Magnetic resonance spectroscopic imaging studies[95,96] have demonstrated reductions in the *N*-acetyl aspartate to creatine ratio in the cerebellum of ET cases compared with controls. *N*-Acetyl aspartate is an amino acid that is present in the cytosol of neurons and is a marker of neuronal integrity. Reductions in *N*-acetyl aspartate (often expressed as a ratio to brain creatine) are a marker of neuronal damage and probable neuronal loss[97,98]. These reductions in the *N*-acetyl aspartate to creatine ratio in the ET cerebellum are consistent with postmortem studies that have documented degenerative changes, including Purkinje cell loss, in the ET cerebellum[79,99]. Hypothesizing that blood harmane concentration in ET cases would be inversely correlated with cerebellar NAA/tCR, a neuroimaging measure of neuronal dysfunction or degeneration, Louis et al.[100] used magnetic resonance spectroscopic imaging to assess 12 ET cases in a pilot study. *A priori*, the neuroanatomic structure of interest was the cerebellar cortex. Secondary regions were the central cerebellar white matter, cerebellar vermis, thalamus, and basal ganglia. Blood concentrations of harmane and another neurotoxin, lead, were also assessed. In a linear regression model that adjusted for age and gender, log blood harmane concentration was a predictor of cerebellar NAA/tCR ($p$ = 0.009) such that every 1 $g^{-10}$/mL unit increase in log blood harmane concentration was associated with a 0.41 unit decrease in cerebellar NAA/tCR. The association between blood harmane concentration and brain NAA/tCR only occurred in the cerebellar cortex; it was not observed in any of the secondary brain regions of interest. Furthermore, the association was specific to harmane and not lead[100]. These results provide preliminary support for the view that increased blood harmane concentration in ET is associated with cerebellar neuronal damage, consistent with the animal study data that harmane and other β-carboline alkaloids produce cerebellar damage[64,69,70,75, 76,77,78]. Of course, this pilot study did not directly measure brain harmane concentration in ET cases, and studies of human postmortem tissue are needed in order to test the association between brain harmane levels and ET directly.

## Other Possible Biological Correlates of Elevated Harmane in ET

In a number of studies of ET patients, additional biological correlates of elevated harmane have been examined in an attempt to explore the wider biological effects of this toxin. In the first study[101], the association between elevated blood harmane and impaired olfaction was explored. Olfactory dysfunction occurs in a variety of diseases, including patients with cerebellar diseases[102,103], and there is evidence that the cerebellum itself may play a role in central olfactory processing[102,103,104,105]. Olfactory dysfunction has also been reported in patients with ET in some studies[106]. As noted above, the pathophysiology of ET is not well understood, although recent postmortem studies indicate the presence of degenerative changes in the cerebellum[24,25]. β-Carboline alkaloids have been shown to produce marked cerebellar damage in exposed laboratory animals in several studies[70,77]. To further test the model that harmane, through cerebellar toxicity, leads to ET, Louis et al.[101] hypothesized that they would find a correlation between blood harmane concentrations and smell test scores in ET cases (i.e., higher blood harmane concentration would be associated with greater olfactory dysfunction) and furthermore, that such a correlation would not be detected among a group of controls. In that study of 83 ET cases and 69 controls, they indeed found that higher blood harmane concentration was correlated with lower smell test scores in ET cases (rho = –0.46, $p < 0.001$); however, in controls, higher blood harmane concentration was not correlated with lower smell test scores (rho = 0.12, $p = 0.32$)[101].

In a second study, Louis et al.[107] examined associations between harmane and cancer. Many of the heterocyclic amines are also mutagens and are linked with several types of cancer (especially colon and prostate)[59,108,109]. Harmane itself is not mutagenic, yet it exerts comutagenic activity both in bacterial and mammalian cells[110,111]. Harmane is a comutagen and a high blood concentration could conceivably predispose an individual to cancer. As noted above, blood harmane concentrations also seem to be elevated in initial studies of ET patients[93,94]. Louis et al.[107], therefore, tested the hypothesis that particularly high blood harmane concentrations might be found among patients with both conditions (i.e., ET and cancer). In a study of 147 ET cases and 187 controls, log blood harmane concentration was highest in ET cases with cancer (0.87 ± 0.68 $g^{-10}$/mL), intermediate in ET cases without cancer (0.54 ± 0.60 $g^{-10}$/mL), and lowest in controls (0.43 ± 0.69 $g^{-10}$/mL) ($p = 0.009$)[107], providing some support for this hypothesis.

Each of the studies discussed above examined additional biological correlates of elevated harmane in ET, further strengthening the notion that harmane exposure has a biological effect in this group of patients.

## What is the Mechanism for the Elevated Blood Harmane Level in ET?

The mechanism for the putative elevation in blood harmane level in ET has not been established. A number of possibilities exist, including increased dietary intake, impaired ability to metabolize harmane (likely genetic), increased endogenous production of harmane (likely genetic), or a combination of these. Data are limited, but the evidence for or against each hypothesis will be reviewed.

As noted above, harmane is found in high concentrations in cooked meats, although there are no data relating the quantity and type of meat ingested with specific postprandial blood harmane levels. Looking at dietary differences, in 2005, Louis et al.[112] compared the levels of daily animal protein consumption in a sample of 106 ET cases and 161 controls. Total daily animal protein consumption was similar in the two groups (50.2 ± 19.6 vs. 49.4 ± 19.1 g/day, $p = 0.74$). However, the study used a standardized food frequency questionnaire[113] that included only a small number of questions to assess meat consumption. In a follow-up study[114], a more detailed meat consumption questionnaire was utilized. In the follow-up study[114], total meat consumption was greater in men with ET vs. men without ET (135.3 ± 71.1 vs. 110.6 ± 80.4 g/day, $p = 0.03$), but not in women with ET vs. without ET (80.6 ± 50.0 vs. 79.3 ± 51.0 g/day, $p = 0.76$). Male ET cases had a higher odds of being in the highest vs. lowest quartile of current meat consumption than did male controls (unadjusted odds ratio [OR] = 9.29, 95% confidence interval [CI] = 2.29–37.64, $p = 0.002$, adjusted OR = 21.36, 95% CI = 3.52–129.51, $p = 0.001$). The study provided preliminary evidence of a dietary difference between males with ET vs. males without ET[114], raising the question whether this possible dietary difference, through harmane consumption, could be linked with ET. Yet the data are very preliminary. First, if some males were eating more meat and developing ET, then one might expect a higher prevalence of ET among males, yet most population-based prevalence studies point to a similar prevalence of ET among males and females rather than a higher prevalence among males[22]. Second, in a population-based study of Hindus in India, many of whom are vegetarian, the prevalence of ET was low (0.35%), but it was similar to other studies of nonvegetarians that used a two-phase approach (i.e., a screen followed by an examination) to capture ET cases[115]. Third, as noted above, cooking meat at higher temperatures for longer periods produces higher harmane concentrations in the meat[90]. Hence, one might expect ET cases to have been eating meat that was more well done than controls. In the dietary study[114], level of meat "doneness" was assessed and was similar in male ET cases and male controls, as well as female ET cases and controls.

An alternative possibility is that the elevated harmane in ET is due to an inherited reduction in the capacity to metabolize harmane. In the replicate sample in New York, Louis et al.[94] stratified ET cases based on family history of ET. The ET cases with a family history of ET had the highest blood harmane concentration, followed by ET cases without a family history of ET, and then finally the controls, who had the lowest concentration ($p = 0.026$). This observation that blood harmane concentration was highest in familial ET cases provided some support for the notion that genetic/metabolic factors are of mechanistic importance[94]. As noted above, the metabolic pathway for harmane is not fully known, although it is probable that it is converted by the liver cytochrome P-450 system to harmine through simple hydroxylation and then methylation steps[60]. To further explore the genetic/metabolic hypothesis, Louis et al. (submitted for publication) recently examined data on blood concentrations of harmane and harmine in more than 500 individuals, comprised of three groups: familial ET, sporadic ET, and controls. The investigators hypothesized that defective metabolic conversion of harmane to harmine might underlie the observed elevated harmane concentration in ET, and therefore expected to find a higher harmane to harmine ratio in familial ET than in sporadic ET or controls. Indeed, the harmane/harmine ratio was highest in familial ET (46.7 ± 140.4), intermediate in sporadic ET (28.3 ± 108.1), and lowest in controls (13.5 ± 50.3) ($p = 0.03$). These findings lend some support to the possibility that the basis for the elevated blood harmane concentration in ET is a genetically driven reduction in harmane metabolism.

A third possibility is that endogenous production of harmane is increased in ET cases. Unfortunately, the relative contributions of exogenous vs. endogenous harmane have not been determined in normal humans nor have they been determined among humans with certain disease states (e.g., ET). There are at present no data to test this possibility directly.

A combination of these factors is also a possibility (e.g., increased dietary exposure as well as decreased metabolic turnover in ET cases).

## Limitations of Current Studies

As noted above, studies of harmane and its putative association with ET have been intriguing. Additional work is required in order to establish and then explore these links further. Although elevated harmane concentrations have been observed in blood samples of ET cases, at this time it is not known whether brain concentrations of this neurotoxin are elevated in ET. Second, current data were derived from studies of ET cases ascertained from a single center and they need to be reproduced elsewhere. Third, one study found elevated blood harmane concentrations in 36 patients with Parkinson's disease[116], which raises the question as to whether elevated blood harmane is specific to ET or is merely a more global marker of neurological illness. Addressing these additional questions would further delineate the associations between ET and this neurotoxin. One final caveat is as follows: the tremor-producing and neurotoxic properties of the β-carboline alkaloids have been presented in this paper. Some of these alkaloids may also have neuroprotective properties (e.g., 9-methyl-β-carboline has been shown to be protective of cultured dopaminergic neurons)[117]. Whether such properties are relevant to tremor disorders is unknown, but worthy of future consideration.

## SUMMARY AND CONCLUSIONS

ET is a common and widespread neurological disease whose etiology is likely to be the result of both genetic and environmental factors. Heterogeneity of pathophysiology, which is becoming evident in postmortem studies[79,118,119], suggests that this disease itself may actually be more than one disease. Hence, establishing an etiological role for a particular neurotoxin is even more challenging, particularly as neurotoxins may be important for some, but not other, forms of the disease. Furthermore, while a neurotoxin by itself could be sufficient to cause a neurological disease, another possibility is that the toxin merely increases the propensity for developing the disorder. For example, a toxin might set in motion a set of biological changes (e.g., Purkinje cell loss), which then increase the exposed individual's sensitivity to a second toxic exposure (e.g., another neurotoxin) or a second nontoxic exposure (e.g., an increased genetic susceptibility to Purkinje cell loss).

Using the commonly cited value of 50% for the percentage of ET cases that occur on a familial basis[30,31] and given its population prevalence of 4.0% beginning at age 40 and older[23], this then suggests that approximately 2.0% of the population aged ≥40 years has a nonfamilial form of ET[23,32]. Environmental etiologies are likely to be of prime importance not only in these cases, but they could also play a role in familial cases through gene-environment interactions. Yet environmental correlates for ET are just beginning to be explored. Initial studies of harmane are of interest, and the role that harmane might play in the etiology of ET deserves additional study using replicate samples of ET cases and brain tissue from such cases. If, indeed, harmane is an etiological agent of importance in ET, from a public health vantage point, one potential means to reduce or prevent the occurrence of ET might be to induce hepatic drug-metabolizing enzymes in the liver P450 system in at-risk individuals (e.g., individuals with a family history of ET).

## ACKNOWLEDGMENTS

Elan D. Louis was funded by R01 NS39422, P30 ES09089, and RR00645 (General Clinical Research Center) from the National Institutes of Health (Bethesda, MD). Wei Zheng was funded by R01 NS39422, R01 ES008146, and R21 ES017055 from the National Institutes of Health (Research Triangle, NC). The National Institutes of Health played no role in the study design, the collection of data, the analysis and interpretation of data, the writing of the report, or in the decision to submit the paper for publication.
